# 2× genomes - depth does matter

**DOI:** 10.1186/gb-2010-11-2-r16

**Published:** 2010-02-09

**Authors:** Michel C Milinkovitch, Raphaël Helaers, Eric Depiereux, Athanasia C Tzika, Toni Gabaldón

**Affiliations:** 1 Laboratory of Artificial and Natural Evolution (LANE), Department of Zoology and Animal Biology, Sciences III, 30, Quai Ernest-Ansermet, 1211 Geneva 4, Switzerland; 2 Department of Biology, Facultés Universitaires Notre-Dame de la Paix, rue de Bruxelles 61, 5000 Namur, Belgium; 3 Department of Evolutionary Biology and Ecology, Université Libre de Bruxelles, Av. F.D. Roosevelt, 50, B-1050 Brussels, Belgium; 4 Centre de Regulació Genòmica (CRG), Dr. Aiguader, 88, 08003 Barcelona, Spain

## Abstract

**Background:**

Given the availability of full genome sequences, mapping gene gains, duplications, and losses during evolution should theoretically be straightforward. However, this endeavor suffers from overemphasis on detecting conserved genome features, which in turn has led to sequencing multiple eutherian genomes with low coverage rather than fewer genomes with high-coverage and more even distribution in the phylogeny. Although limitations associated with analysis of low coverage genomes are recognized, they have not been quantified.

**Results:**

Here, using recently developed comparative genomic application systems, we evaluate the impact of low-coverage genomes on inferences pertaining to gene gains and losses when analyzing eukaryote genome evolution through gene duplication. We demonstrate that, when performing inference of genome content evolution, low-coverage genomes generate not only a massive number of false gene losses, but also striking artifacts in gene duplication inference, especially at the most recent common ancestor of low-coverage genomes. We show that the artifactual gains are caused by the low coverage of genome sequence *per se *rather than by the increased taxon sampling in a biased portion of the species tree.

**Conclusions:**

We argue that it will remain difficult to differentiate artifacts from true changes in modes and tempo of genome evolution until there is better homogeneity in both taxon sampling and high-coverage sequencing. This is important for broadening the utility of full genome data to the community of evolutionary biologists, whose interests go well beyond widely conserved physiologies and developmental patterns as they seek to understand the generative mechanisms underlying biological diversity.

## Abstract 2

The use of low coverage genomes in comparative evolutionary analyses skews estimates of gene gains and losses.

## Background

In the context of investigating correlations between genome and phenotype evolution, describing the evolution of genome content (in terms of protein-coding genes) should theoretically be straightforward given the increasing number of available sequenced genomes and of large-scale expression studies, accompanied by a constantly growing number of software and databases for better integration and exploitation of this wealth of data. However, this endeavor of mapping gene gains (including duplication events) and losses suffers from the lack of explicit phylogenetic criteria in analytical tools, and the overemphasis, in genome sequencing programs, on detecting conserved genome features.

The first problem relates to the fact that many of the methods and databases available for identifying duplication events and assessing orthology relationships of genetic elements among genomes avoid the heavy computational cost of phylogenetic trees inference and the difficulties associated with their interpretation, even though phylogeny-based orthology/paralogy identification is widely accepted as the most valid approach [1-4]. Recently, however, the problem has been largely recognized and increasingly addressed by the comparative genomics community. For example, ENSEMBL [5,6] and the 'phylome' approach [7,8] are automated pipelines in which orthologs and paralogs are systematically identified through the estimation of gene family phylogenetic trees. Furthermore, the recently developed MANTiS relational database [9] integrates phylogeny-based orthology/paralogy assignments with functional and expression data, allowing users to explore phylogeny-driven (focusing on any set of branches), gene-driven (focusing on any set of genes), function/process-driven, and expression-driven questions in an explicit phylogenetic framework. Such application systems should help in investigating whether the gene duplication phenomenon is generally relevant to adaptive evolution (that is, beyond the classical examples of, for example, globins, olfactory receptors, opsins, and transcription factor diversifications), and might even help in understanding the causal relationships between genome evolution and increasing phenotypic complexity. However, the efficiency of these analytical tools inescapably depends on the amount and quality of the available genome sequence data. This leads us to the second, more pervasive problem of biases in whole genome sequencing program strategies.

**Figure 1:**
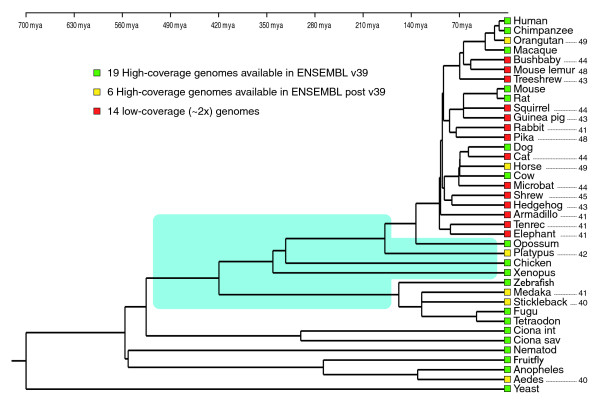
**Phylogeny among the 39 species whose genomes are available in version 49 of the ENSEMBL database**. Approximate age of nodes is from [34]. The area shaded in blue indicates long branches in vertebrates that should preferentially be interrupted by the sequencing of additional full genomes. Levels of sequence coverage are color-coded and numbers on the right of the tree indicate the ENSEMBL version in which the species appeared for the first time in the gene family trees. Mya, million years ago.

Sequencing and analyzing the complete genome of a eukaryotic species is a formidable and challenging task, and the human genome project [10,11] will probably remain a landmark in the history of science. Incentives for sequencing genomes of non-human species mirror historical motives for selecting laboratory model species: the potential power of these species for understanding human biology and generating biomedically relevant data. This criterion has generated a striking taxonomic bias in the choice of model species and sequencing projects [12]. For example, only 3% of full-genome sequencing projects use the localization of the corresponding species in the tree of life as a primary motivation [13]. As a result, prominent databases like ENSEMBL [14], which generates and maintains automatic annotation of selected eukaryotic genomes, included 25 mammalian and 5 teleost fish genomes, but only one bird, one amphibian, and no reptile in its version 49 (Figure 1).

One major explicit goal of genome sequencing projects is that comparisons of the human genome with those of other eukaryotes allow detection of coding and non-coding conserved (hence, likely functional) elements in the human genome. Importantly, the statistical power of such comparisons depends on the sum of branch lengths of the phylogenetic tree among the species used [15]. However, it is likely that a significant proportion of these possibly biomedically relevant conserved features are recent and thus specific to relatively shallow branches (for example, mammals, eutheria, primates) rather than common to all eukaryotes. In that case, the only way to increase statistical power is to increase the number of sequenced genomes for species belonging to the monophyletic group defined by the relevant shallow branch. This realization has motivated the development of the 'Mammalian Genome Project' [16] aiming at sequencing the genome of multiple placental mammals with a low mean coverage of 2×. The sequenced species were chosen to maximize the ratio [Sum of branch lengths within mammals]/[Number of genomes sequenced]. Note that the decision to choose the placental mammal branch is somewhat arbitrary: there is no *a priori *reason to believe that there are more (or more important) Eutherian-specific than, for example, Therian-specific biomedically relevant conserved features, and sequencing a few well-chosen marsupial species would have generated more cumulative branch length for less species. However, this decision might have been motivated by the facts that using a shallower branch will facilitate annotation of the newly sequenced genomes and that some of the chosen species are laboratory model species.

We think that the emphasis on searching for evolutionary conservation - hence, the decision to prefer 24 low-coverage (2×) genomes to, for example, 6 genomes at 8× coverage, hurts more general endeavors, such as the mapping of gene gains and losses in the evolution of eukaryotic genomes. Although the inherent limitations associated with low coverage genome analyses are recognized [15], their impact on understanding differences among organisms (rather than similarities) has not been quantified.

Here, we evaluate the impact of low-coverage genomes on inferences pertaining to gene gains and losses when analyzing the mode and tempo of eukaryote genome evolution through gene duplication. Such assessments are important for broadening the utility of full genome data to the community of evolutionary biologists, whose interests go well beyond widely conserved physiologies and developmental processes/patterns as they seek to understand the generative mechanisms underlying biological diversity.

## Results and Discussion

On the basis of the 38 metazoan genomes (longest splice-variant of each protein-coding gene) available in version 49 of the ENSEMBL database (that is, six primates, one tree shrew, four rodents, two lagomorphs, two carnivores, one perissodactyl, one cetartiodactyl, one bat, two insectivores, one xenarthran, two afrotherians, one marsupial, one monotreme, one bird, one amphibian, five teleost fishes, two urochordates, one nematode, and three insects), and using the baker's yeast as an outgroup, we used MANTiS version 1.0.15 [17] to generate two datasets including information on the presence/absence of genes. The first dataset ('families only') contains one character for each single (species-specific) gene and for each protein family (that is, only *de novo *gains are considered), whereas in the second dataset ('with duplications'), a new character was additionally created for each duplication event, such that each protein family is represented by several characters. Additional details are given in [9]. To investigate the influence of low-coverage (2×) genomes on inferred genome evolutionary patterns, we also generated with MANTiS the corresponding datasets using versions 39 to 48 of ENSEMBL (Figure 1) and the human phylome [8], available at [18]. The ENSEMBL v39 archive database includes 18 metazoan species with 7 placental mammal genomes of coverage >4 (except for the rhesus macaque, *Macaca mulatta*), whereas subsequent versions include an increasing number of low mean coverage (2×) genomes (v49 includes 38 metazoan species with 24 placental mammal genomes, of which 14 are of 2× mean coverage). The PhylomeDB database uses only high-coverage genomes and an improved phylogenetic pipeline that includes alignment trimming, branch-length optimization, evolutionary model testing, and maximum likelihood and Bayesian phylogeny inference (see Materials and methods for details).

**Figure 2:**
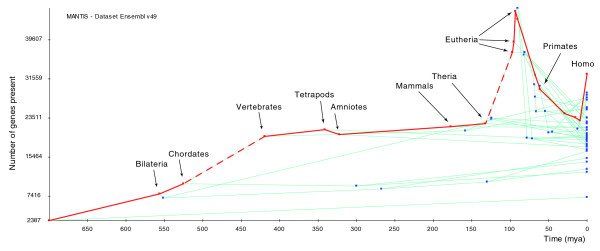
**Increase of genome size through evolutionary time for all lineages of the tree in Figure 1**. The red line indicates the lineage leading to the human genome. The left and right dashed lines indicate the branches leading to the vertebrate and the first eutherian node, respectively. Mya, million years ago.

**Figure 3:**
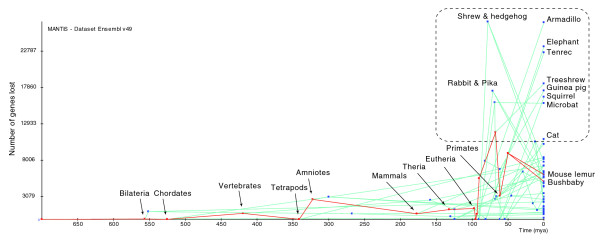
**Mapping of losses through evolutionary time for all lineages of the tree in Figure 1**. The red line indicates the lineage leading to the bushbaby and mouse lemur genomes. The 12 other low-coverage genomes are framed (dashed line). Mya, million years ago.

Using MANTiS, we mapped gains and losses of characters on the species phylogeny best supported by previous phylogenetic analyses [19-21]: gains are assigned directly from the topology of gene family trees whereas the most likely positions of gene losses are estimated using a maximum likelihood function (see Materials and methods). These character mapping analyses show that acquisition of new genes is an important, continuous, and general phenomenon and explains part of the increase in genome size during evolution. Plotting, for all species lineages, genome size - in terms both of number of predicted gene counts (Figure 2) and sum of gene length (data not shown) - against evolutionary time indicates that the rate of gains on the vertebrate linage (Figure 2, left dashed line) is particularly high, a result explained by the two rounds of whole genome duplication that occurred at the dawn of vertebrate evolution [8,22]. This high increase in gene number is exceeded, however, on the first eutherian (true mammals) branch (Figure 2, right dashed line), a particularly spectacular result given the much smaller length (in terms of evolutionary time) of the eutherian compared to the vertebrate branch. Equally striking is the reduction in genome size for all taxa after the three first basal eutherian branches (Figure 2). However, it is likely that most of the subsequent massive gene losses, after the eutherian peak in gene gains, are artifacts caused by low quality genomes. Indeed, plotting the number of gene losses against evolutionary time (Figure 3) indicates that 12 of the 14 low-coverage genomes in v49 of ENSEMBL are associated with the largest number of losses in the corresponding terminal branches, or in the most recent common ancestor of pairs of these taxa (for example, the insectivore or the lagomorph nodes). The two remaining low coverage genomes (bushbaby and mouse lemur) suffer less artifactual losses probably because a large number of false losses have been assigned to deeper ancestors of these two species and because annotation of these genomes was greatly facilitated by the use of the high-quality human genome.

**Figure 4:**
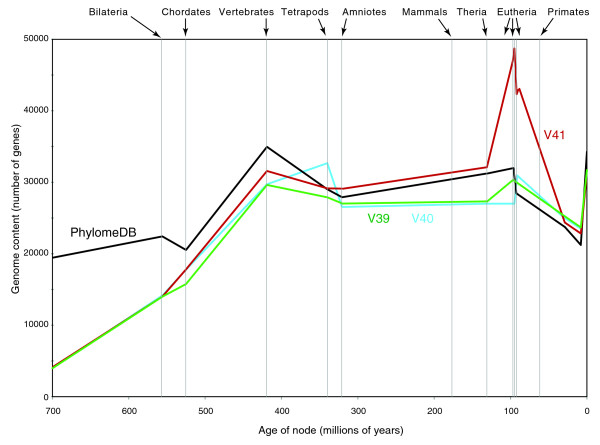
**Inference of increased gene content through evolutionary time for the lineage leading to the human genome**. The analysis is performed with versions 39, 40, and 41 of the ENSEMBL database as well as with the human phylome (PhylomeDB, as of December 2008). The inclusion of low-coverage genomes (appearing for the first time in ENSEMBL v41 gene trees) generates an artifactual peak of gene gains at the eutherian nodes. Note that the first four low-coverage genomes (rabbit, elephant, tenrec, and armadillo) were added in the sequence database, but not the gene trees, of version 40.

We used MANTiS to map genome size against evolutionary time (for the lineage leading to human) for various versions of the ENSEMBL database and for the Phylome database. These analyses indicate that the addition of low-coverage genomes (appearing in version 41 of ENSEMBL) generate the high, and probably artifactual, rate of gene gains in the first eutherian branches, whereas the presence of an increased rate of gene gains in the vertebrate branch is robust to the removal of low-coverage genomes (Figure 4).

**Figure 5:**
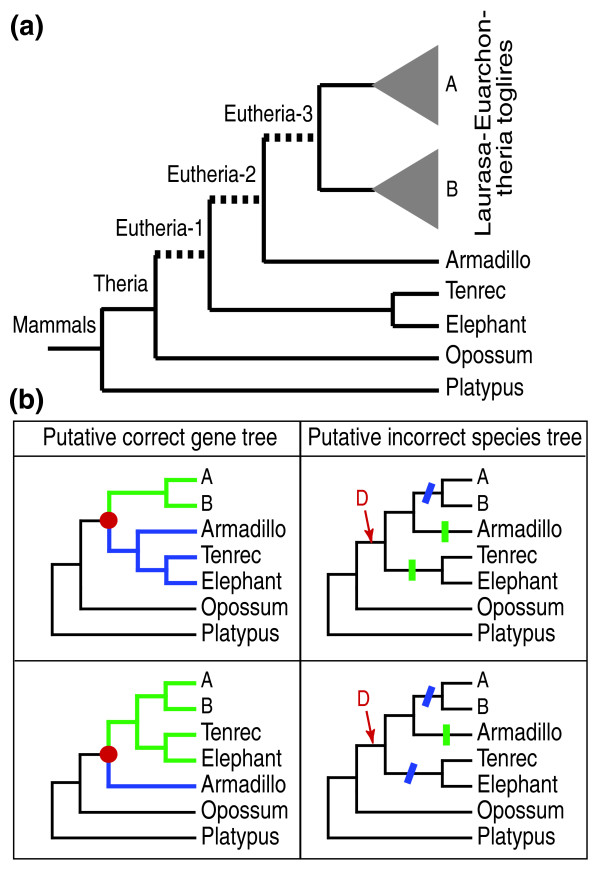
**Possible artifactual gains and losses due to reconciliation between a correct gene tree and an incorrect species tree**. **(a) **Addition of the armadillo and afrotherian (elephant and tenrec) genomes in version 41 of ENSEMBL cut the eutherian branch in three parts; **(b) **If the species tree (right column) is incorrect, reconciliation with correct gene trees (two alternative topologies are given) will generate false duplication events (red dot on gene tree and 'D' on species tree) in the first eutherian branch followed by losses (vertical green bars for loss of the green duplicate, oblique blue bars for loss of the blue duplicate) in various branches.

**Figure 6:**
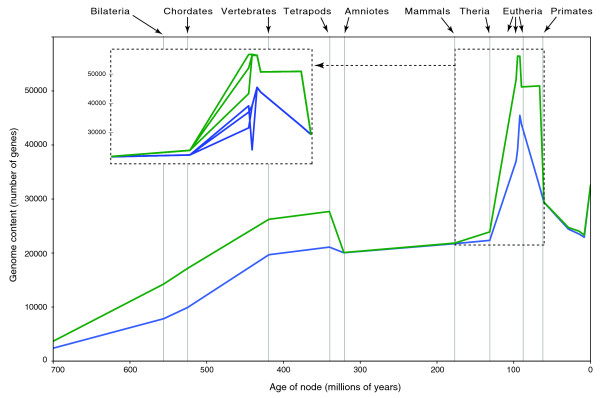
**Mapping of genome content using tree reconciliation performed by us (green) and ENSEMBL (blue)**. The differences between the two curves are due to differences between the species trees used: for example, we group *Caenorhabditis elegans *(nematodes) with arthropods (in the clade Ecdysozoa [35]) whereas it is positioned at the base of Bilateria in the species tree used by ENSEMBL. The inset shows the different mapping generated when using the tree alternative phylogenies among Afrotheria, Armadillo, and the remaining eutherians (groups A and B in Figure 5).

Although the interrupted and missing genes in 2× coverage genomes are likely to generate false losses (Figure 3), they have no obvious *a priori *reason to cause an artifactual increase in duplication events in deeper branches. Similarly, although errors in draft genomes can cause misassemblies and unmerged overlaps - hence, causing errors in the orthology assignment of genes through false positives (artificial duplications) - the phenomenon should not specifically impact the three first eutherian branches more than shallower branches (low coverage genomes are distributed all across the eutherian tree). Note that the eutherian branch, as defined in version 39 of ENSEMBL, is cut in three parts in subsequent versions of ENSEMBL by the addition of the Afrotheria (elephant and tenrec) and the Xenarthra (armadillo) lineages (Figure 5a). A possible explanation for the artifactual peak of gains in the eutherian branches would be that the supposedly true phylogenetic position of Afrotheria and Xenarthra is incorrect: true gene tree versus wrong species tree reconciliation would then generate false duplication events in the first eutherian branch followed by losses (Figure 5b). However, mapping gains and losses in MANTiS after implementing the three possible topologies among the outgroup, Afrotheria, Xenarthra, and Laurasiatheria plus Euarchontoglires did not remove the artifactual peak of gains in the eutherian branches (Figure 6), although the use of the different species trees generated a different distribution of these changes among the three eutherian branches (Figure 6, inset). We performed again reconciliation of all ENSEMBL gene trees with the species phylogeny in which nematodes and arthropods form a monophyletic group. This generated some differences in gains and losses mapping in the base of the tree but did not remove the artifactual peak in the eutherian branches (green curve in Figure 6).

An alternative explanation for the artifactual peak of gene gains in the eutherian branches would be the mirror situation: correct species tree but incorrect gene trees. To test this hypothesis, we first verified whether, in 2× genomes, the mean sequence coverage of genes inferred as duplicated in the three first eutherian branches (version 49) is lower than the mean sequence coverage of genes inferred as duplicated elsewhere in the species tree. As the sequence coverage, nucleotide by nucleotide, or gene per gene, is (to our knowledge) not publicly available, we counted the number of ambiguities in each protein sequence of each species and found that 2× genomes exhibit higher mean proportions of ambiguities, ranging from 9.11% (*Ochotona princeps*) to 15.46% (*Dasypus novemcinctus*), compared to 0 to 0.24% in high coverage genomes. However, we did not observe a higher mean proportion of ambiguities (for neither 2× nor high-coverage genomes) in genes inferred as duplicated in the three first eutherian branches than in genes inferred as duplicated elsewhere in the species tree.

**Figure 7:**
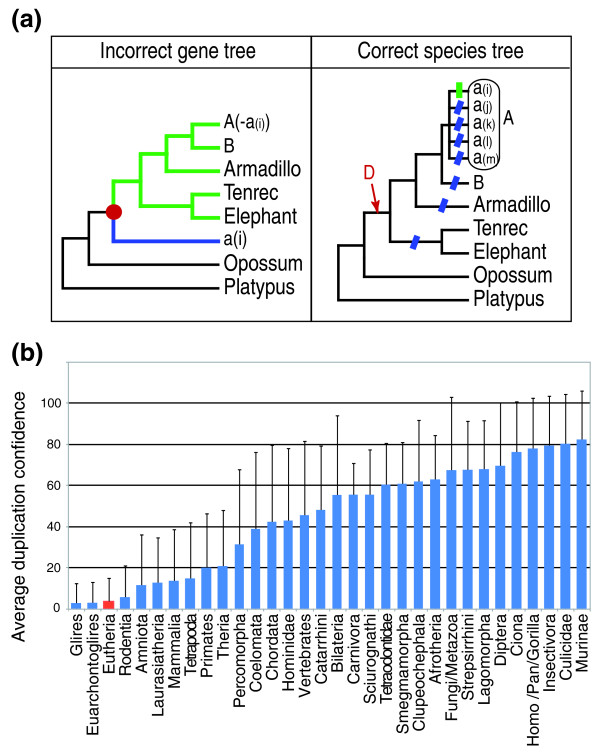
**Possible artifactual gains and losses due to reconciliation between an incorrect gene tree and a correct species tree**. **(a) **Gene trees with a single eutherian species (*a*_*(i)*_) on one side of a duplication node (red dot) and several species on the other side (especially if *a*_*(i) *_is absent from that side) are highly suspicious (Table 1). Such an incorrect gene tree will gen-erate one false duplication on the basal eutherian lineage followed by multiple false losses (vertical green and oblique blue bars). **(b) **Average duplication confidence (and standard de-viation) for all duplication nodes on all 26,467 gene trees from the ENSEMBL version 49 da-tabase. The eutherian node is highlighted in red.

One could argue that many of the artifactual gains in the eutherian nodes might not be caused by the low coverage of 2× genomes *per se *but are rather simply the product of increased taxon sampling in a very biased and small portion of the species tree: increasing the number of mammalian species reduces the lengths of already short branches, hence increasing the risk of misplacing at least one lineage in gene trees and generating false gains and subsequent false losses. For example, any duplication node labeled 'Eutheria' with a single 'orphan' species on one side of the duplication node (blue branch in Figure 7a) is suspicious as it implies one duplication event in the basal eutherian branch and multiple losses in shallower branches. The reality of the duplication event is even more questionable when the orphan species is absent from the upper side of the duplication node (green subtree in Figure 7a) as it requires reciprocal complementary gene losses (a quite unlikely phenomenon indeed). We screened all 26,467 trees in the ENSEMBL database and found that 2× genomes contribute significantly more (0.005 <* P*-value < 0.016; one-tail Mann-Whitney test) than good quality genomes to such suspicious topologies (Table 1). Note also that the four species highest in the list (shrew, hedgehog, pika, and guinea pig) are far from the base of the eutherian tree and are therefore unlikely to be represented by orphan sequences. Statistical significance is not due to the basal Afrotherian and armadillo taxa as removing these species from the list even reduces the *P*-values to 0.001 to 0.007. Finally, for assessing the validity of inferred duplication nodes, we used the species-overlap score of all 115,451 duplication nodes (in the 26,467 ENSEMBLE trees) defined as the fraction of shared species over the total of species in post-duplication nodes [8] (equivalent to the 'duplication consistency score' in [1]). Figure 7b indicates that duplications at the eutherian node exhibit one of the three worst confidence values (mean ± standard deviation = 3.7 ± 11.5) among all nodes in the species phylogeny.

**Table 1:** Number of dubious duplications at the eutherian node involving various species as 'orphans'

Species	Isolated sp*(i)*	sp*(i) *versus >5	sp*(i) *versus >5 - no sp*(i)*	sp*(i) *versus >10	sp*(i) *versus >10 - no sp*(i)*
2× coverage genomes					
*Sorex araneus*	621	591	581	563	557
*Erinaceus europaeus*	565	531	523	498	493
*Ochotona princeps*	376	343	337	308	302
*Cavia porcellus*	344	308	302	280	274
*Echinops telfairi*	272	246	235	237	227
*Myotis lucifugus*	192	168	156	146	137
*Tupaia belangeri*	192	159	150	132	126
*Spermophilus tridecemlineatus*	182	142	140	110	108
*Oryctolagus cuniculus*	149	114	108	93	88
*Otolemur garnettii*	125	100	97	79	76
*Loxodonta africana*	109	77	64	70	59
*Microcebus murinus*	112	90	86	62	59
*Felis catus*	98	73	70	57	54
*Dasypus novemcinctus*	15	6	0	4	0
					
High-coverage genomes					
*Bos taurus*	200	168	131	148	112
*Canis familiaris*	156	120	94	93	71
*Mus musculus*	129	100	94	75	70
*Equus caballus*	134	110	78	86	58
*Rattus norvegicus*	73	52	37	37	28
*Pongo pygmaeus*	34	25	21	17	14
*Macaca mulatta*	62	44	24	28	14
*Pan troglodytes*	24	13	7	10	5
*Homo sapiens*	14	7	5	4	3
					
One-tail Mann-Whitney test					
*P*-value	0.016	0.016	0.005	0.014	0.005

See Figure 7 and text for details. Different columns correspond to cases where one side of a duplication at the eutherian node involved: an orphan species (isolated sp(*i*)), or one orphan species versus more than five (sp(*i*) versus >5) or more than ten (sp(*i*) versus >10) species on the other side of the duplication. The column labeled with 'no sp(*i*)' indicates the cases where the orphan species is absent from the other side of the duplication node (this requires perfect reciprocal complementary gene losses and, hence, corresponds to a species-overlap score of zero). Values are sorted according to the last column.

**Figure 8:**
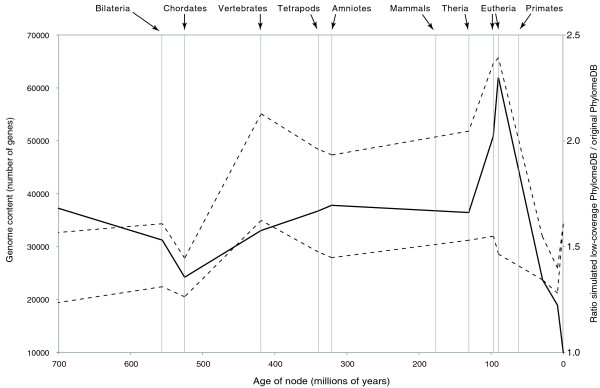
**Simulations of low-coverage genomes and their impact on gene content inference through evolutionary time**. The analysis is performed with the original human phylome (PhylomeDB, lower dashed line) and a simulated low-coverage PhylomeDB (upper dashed line) in which stretches of ambiguous sequences have been introduced in the protein sequences of three of the seven eutherian species. The transformation of these high-coverage genomes into simulated low-coverage genomes generates artifactual gains all across the species tree, but more acutely so at the basal eutherian nodes (the plain line, and secondary axis, indicates the ratio of genome content between the simulated low-coverage PhylomeDB and the original PhylomeDB).

To test our hypothesis, we used large-scale simulations to evaluate the impact of reducing sequence quality on gene tree and duplication inferences. Starting from the high-coverage genomes included in the phylomeDB [7], we randomly introduced continuous stretches of ambiguous sequences in the protein sequences of three eutherian species, *Pan troglodytes*, *Mus musculus *and *Bos taurus*, according to a distribution approximating that observed in real low-coverage sequences. All sequences were then re-aligned and all 19,361 gene trees were reconstructed and analyzed in the same way as the non-perturbed PhylomeDB dataset. Figure 8 indicates that our hypothesis is verified: the introduction of ambiguities in three of the seven eutherian species generates errors in the inference of gene trees that, in turn, produce spurious duplications events. These artifacts are distributed in various places in the species tree, but the most impacted nodes are clearly the basal eutherian lineages (Figure 8).

## Conclusions

We argue that the phylogenetic distribution of species for which so-called 'full genome sequences' are available, as well as the coverage of these genomes, are key parameters that have not been given enough appreciation: it will remain exceedingly difficult to differentiate artifacts from true changes in modes and tempo of genome evolution until better homogeneity in both taxon sampling and high-coverage sequencing is achieved. For example, the groups of Amphibia (frogs, toads, salamanders, newts, and caecilians) or Reptilia (turtles, lizards, crocodiles, and birds) exhibit larger diversities than mammals but have long been represented in major databases such as ENSEMBL by a single species (*Xenopus tropicalis*, and *Gallus Gallus*, respectively) at the tip of a very long branch. The recent inclusion (since ENSEMBL v53) of the high-coverage genome sequences from the green anole lizard (*Anolis carolinensis*) and zebra finch (*Taeniopygia guttata*) are, in this respect, very important for improved mapping reliability of genome content evolution in the amniote tree. Similarly, including some of the missing major animal lineages (for example, Lophotrochozoans such as annelids, molluscs, and flat worms) is crucial if reliable analysis is to be extended to the whole group of Metazoa. However, major artifacts in gene gains and losses (and possibly others that we did not uncover here) will remain until all low-coverage genomes are promoted to high coverage. Note that very recent (generally species-specific) duplications will remain very difficult to differentiate from parental alleles even in high-coverage genomes.

Obviously, the artifactual gains and losses of duplicates discussed here are problematic only for a subset of comparative genomic analyses. For example, these artifacts are of low relevance for the specific and significant purpose behind the initial production of low-coverage genomes: detecting conserved genome features [15]. Furthermore, these artifacts had little impact on analyses that uncovered historical constraints in gene expression [23], despite these analyses requiring the determination of the first appearance of genes and duplicates in the species phylogeny. However, artifacts in mapping of genome content evolution will likely mislead many users who access genomic databases, possibly resulting in a wave of unreliable analyses.

Fortunately, the tremendous drop in sequencing costs brought about by next generation sequencing platforms (for example, [24,25]) allows the comparative genomics community to contemplate the possibility of sequencing, in the coming decade, hundreds or even thousands of complex genomes spanning a wide phylogenetic diversity (for example, [26]). We, however, urge the community to go for quality rather than for quantity: high-coverage should be a compulsory requirement in these large genome sequencing projects such that genome content evolution, as well as coding and non-coding sequence changes, can be reliably inferred for a vastly improved understanding of genome evolution.

## Materials and methods

### PhylomeDB data

As an alternative to ENSEMBL trees, we used data from the human phylome [8] available through the PhylomeDB database [7]. The pipeline used to reconstruct the human phylome is described in more detail elsewhere [8]. In brief, a database containing all proteins encoded in the 39 eukaryotic genomes (all high coverage) included in the phylome is searched for putative homologs of human proteins by a Smith-Waterman algorithm [27]. Significant hits with an e-value lower than 10^-3 ^and that could be aligned over a continuous region longer than 50% of the query sequence were selected and subsequently aligned with MUSCLE 3.6 [28]. Alignments are trimmed using trimAl 1.0 [29] to remove columns with gaps in more than 10% of the sequences, unless such a procedure removes more than one-third of the positions in the alignment. In such cases the percentage of sequences with gaps allowed is automatically increased until at least two-thirds of the initial columns are conserved. Finally, phylogenetic trees are reconstructed by using maximum likelihood as implemented in PhyML v2.4.4 [30]. In all cases a discrete gamma-distribution model is assumed with four rate categories and invariant sites, where the gamma shape parameter and the fraction of invariant sites are estimated from the data. To avoid model-based biases, protein evolutionary models (JTT, Dayhoff, MtREV, VT and BLOSUM62) are tested to then select the one best fitting the data according to the Akaike information criterion (AIC) [31].

### Gene tree-species tree reconciliation

We used a strict tree-reconciliation algorithm [32] as implemented in ETE [33]. In this case, every gene tree is compared to the topology of a given species tree by comparing the specific sets of species contained by all tree splits. The strict reconciliation algorithm maps the gene tree onto the species tree and explains any incongruence in terms of the minimal set of duplication and gene-loss events necessary to derive the observed gene tree topology from the one proposed in the species tree. These inferred duplication events are marked on the tree, and orthology and paralogy relations are derived accordingly.

### Simulation of low coverage sequence data

To evaluate the phylogenetic effects of low quality sequence data, stretches of ambiguous sequences where introduced in the protein sequences of three species (*P. troglodytes*, *M. musculus *and *B. taurus*) of the phylomeDB. Continuous stretches of amino acids were substituted by 'X's according to a normal distribution of lengths with mean μ, and standard deviation δ. These parameters were set for each of the three selected species: *P. troglodytes *(μ = 9%, and δ = 3% of the length of the sequence); *M. musculus *(12%, 3%); and *B. taurus *(15%, 3%); that is, according to the range of values we observed in real low-coverage genomes. After introducing the simulated ambiguities, sequences were re-aligned and trees were reconstructed and analyzed in the same way as the non-perturbed PhylomeDB dataset.

## Authors' contributions

MCM and ACT conceived the study. MCM and RH performed genome content (gene gains and gene losses) mapping and duplication analyses. ED provided computing facilities. TG provided the PhylomeDB and perturbed (low-coverage) PhylomeDB raw data, and performed additional tree reconciliation analyses with MCM and RH. MCM, ACT, and TG wrote the manuscript. All authors read and approved the final manuscript.
